# Nitric oxide balances osteoblast and adipocyte lineage differentiation via the JNK/MAPK signaling pathway in periodontal ligament stem cells

**DOI:** 10.1186/s13287-018-0869-2

**Published:** 2018-05-02

**Authors:** Shan Yang, Lijia Guo, Yingying Su, Jing Wen, Juan Du, Xiaoyan Li, Yitong Liu, Jie Feng, Yongmei Xie, Yuxing Bai, Hao Wang, Yi Liu

**Affiliations:** 1 Laboratory of Tissue Regeneration and Immunology and Department of Periodontics, Beijing Key Laboratory of Tooth Regeneration and Function Reconstruction, School of Stomatology, Capital Medical University, Tian Tan Xi Li No.4, Beijing, 100050 People’s Republic of China; 2 Department of Orthodontics, Capital Medical University School of Stomatology, Beijing, People’s Republic of China; 3 Department of Stomatology, Beijing Tiantan Hospital, Capital Medical University, Beijing, People’s Republic of China

**Keywords:** Periodontal ligament stem cells, Nitric Oxide, Osteogenesis, Adipogenesis, JNK/MAPK signaling pathway

## Abstract

**Background:**

Critical tissues that undergo regeneration in periodontal tissue are of mesenchymal origin; thus, investigating the regulatory mechanisms underlying the fate of periodontal ligament stem cells could be beneficial for application in periodontal tissue regeneration. Nitric oxide (NO) regulates many biological processes in developing embryos and adult stem cells. The present study was designed to investigate the effects of NO on the function of human periodontal ligament stem cells (PDLSCs) as well as to elucidate the underlying molecular mechanisms.

**Methods:**

Immunofluorescent staining and flow cytometry were used for stem cell identification. Western blot, reverse transcription polymerase chain reaction (RT-PCR), immunofluorescent staining, and flow cytometry were used to examine the expression of NO-synthesizing enzymes. The proliferative capacity of PDLSCs was determined by EdU assays. The osteogenic potential of PDLSCs was tested using alkaline phosphatase (ALP) staining, Alizarin Red staining, and calcium concentration detection. Oil Red O staining was used to analyze the adipogenic ability. Western blot, RT-PCR, and staining were used to examine the signaling pathway.

**Results:**

Human PDLSCs expressed both inducible NO synthase (iNOS) and endothelial NO synthase (eNOS) and produced NO. Blocking the generation of NO with the NOS inhibitor l-N^G^-monomethyl arginine (l-NMMA) had no influence on PDLSC proliferation and apoptosis but significantly attenuated the osteogenic differentiation capacity and stimulated the adipogenic differentiation capacity of PDLSCs. Increasing the physiological level of NO with NO donor sodium nitroprusside (SNP) significantly promoted the osteogenic differentiation capacity but reduced the adipogenic differentiation capacity of PDLSCs. NO balances the osteoblast and adipocyte lineage differentiation in periodontal ligament stem cells via the c-Jun N-terminal kinase (JNK)/mitogen-activated protein kinase (MAPK) signaling pathway.

**Conclusions:**

NO is essential for maintaining the balance between osteoblasts and adipocytes in PDLSCs via the JNK/MAPK signaling pathway**.**

**Graphical Abstract:**

NO balances osteoblast and adipocyte lineage differentiation via JNK/MAPK signaling pathway
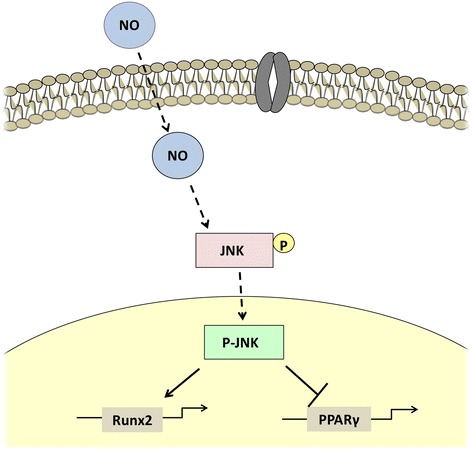

**Electronic supplementary material:**

The online version of this article (10.1186/s13287-018-0869-2) contains supplementary material, which is available to authorized users.

## Background

Periodontitis is one of the most widespread infectious diseases and is characterized by chronic bacterial infection of the supporting structures of the teeth, leading to tooth loss in adults [1]. Stem cell therapy has been shown to be a promising strategy for the treatment of periodontitis [2]. Although many signaling pathways and molecules have been identified that regulate the differentiation of mesenchymal stem cells (MSCs), the precise mechanisms determining the fate of stem cells are unclear [3]. This results in limited clinical translation of stem cell therapy. Understanding the mechanisms underlying the fate of stem cells would be helpful for application in regenerative medicine.

Nitric oxide (NO) is a gaseous radical that is recognized as one of the smallest known bioactive products of mammalian cells. Endogenous NO is primarily generated by NO synthase (NOS) enzymes. Three distinct isoforms of NOS have been identified: neuronal NOS (nNOS), endothelial NOS (eNOS), and inducible NOS (iNOS) [4]. Accumulating evidence suggests that many biological processes are regulated by NO in developing embryos and adult stem cells [5, 6]. The modulation of cell function depends on specific local concentrations of NO, and opposite effects can be observed when low and high levels of NO are compared. NO at physiological concentrations has been shown to promote MSC survival, homing, and differentiation, and NO also maintains the self-renewal potential of neuronal stem cells and their efficacy against ischemic conditions [7]. Moderate levels (100 μM) of NO donor can enhance the survival rate of MSCs via protection from renal ischemic injury after kidney damage [8]. These results highlight the importance of NO as a potential modulator of stem cell therapies.

Periodontal ligament stem cells (PDLSCs) have been shown to possess great potential in periodontal regenerative therapies. It has been suggested that NO mediates the differentiation of PDLSCs into osteoblasts, as demonstrated by an increase in NO production during osteogenic differentiation of PDLSCs [8]. Periodontal ligament (PDL) cells treated with exogenous NO exhibit enhanced osteogenic potential [9, 10]. However, the role of endogenous NO in regulating the fate of PDLSCs, as well as the underlying molecular mechanisms, remain poorly understood. The present study was designed to investigate the effects of NO on the functions of human PDLSCs and the possible signaling pathway underlying the process. Our study found that blocking the production of NO with a NOS inhibitor decreased the osteogenic capacity of PDLSCs but promoted their adipogenic capacity, whereas adding additional physiological levels of NO with sodium nitroprusside (SNP) decreased the adipogenic capacity of PDLSCs but promoted their osteogenic capacity, indicating the importance of NO in balancing the osteogenic and adipogenic potential of human PDLSCs. In addition, we show that NO regulates the differentiation function of PDLSCs through the c-Jun N-terminal kinase (JNK)/mitogen-activated protein kinase (MAPK) signaling pathway.

## Methods

### Cell culture

The study was performed according to an informed protocol for handling human tissue approved by the Research Ethical Committee of Capital Medical University, China (2012-x-53). The method for culturing periodontal ligament stem cells has been described previously [11]. Experiments were performed using the third generation of human PDLSCs.

### Western blot analysis

The protocol has been described previously [12]. Proteins of interest were detected using anti-iNOS (1:1000, Novus), anti-eNOS (1:1000, Abcam), anti-alkaline phosphatase (ALP; 1:1000, Abcam), anti-runt-related transcription factor 2 (Runx2; 1:1000, Abcam), anti-peroxisome proliferator-activated receptor (PPAR)γ (1:1000, Abcam), anti-p-JNK (1:1000, Abcam), or anti-JNK (1:1000, Abcam) antibodies, while β-actin was detected with anti-β-actin antibody (1:2000, Abcam) as a control.

### Flow cytometric analysis

For flow cytometric analysis of iNOS and eNOS expression, PDLSCs were harvested and fixed with 80% methanol and then permeabilized with 0.1% PBS-Tween for 20 min. Cells were then incubated in phosphate-buffered saline (PBS)/10% normal goat serum/0.3 M glycine to block nonspecific protein-protein interactions by the anti-iNOS and anti-eNOS antibodies (Abcam, 1 μg/1 × 10^6^ cells). Fluorescein isothiocyanate (FITC) goat anti-mouse immunoglobulin (Ig)G was used as a secondary antibody (BioLegend, 1 μg/1 × 10^6^ cells). Cells were analyzed with a fluorescein-activated cell sorter (FACS) Calibur flow cytometer (BD Immunocytometry Systems, San Jose, CA, USA).

For flow cytometric analysis of stem cell identification, PDLSCs were harvested and fixed with 80% methanol for 20 min. Fixed cells were incubated in sealing buffer for 30 min and then incubated in 3% bovine serum albumin (BSA)/PBS with anti-CD44, anti-CD45, and anti-CD146 antibodies (Abcam, 1 μg/1 × 10^6^ cells). FITC goat anti-rabbit IgG was used as a secondary antibody (BioLegend, 1 μg/1 × 10^6^ cell). Cells were analyzed with a FACS Calibur flow cytometer (BD Immunocytometry Systems).

### EdU assay for cell proliferation

PDLSCs were seeded in six-well plates (Nunc) and cultured for 2–3 days. The cultures were incubated with EdU solution (1:1000, Invitrogen) for 24 h and stained with Click-iT EdU Flow Cytometry Assay Kits (Invitrogen) according to the manufacturer’s instructions. Cells were analyzed with a flow cytometer (BD Immunocytometry Systems). The number of EdU-positive cells was indicated as a percentage of the total cell number.

### Determination of apoptotic cell percentage

To detect apoptotic cells, we utilized the Annexin V Apoptosis Detection Kit FITC (eBioscience, San Diego, CA, USA) according to the manufacturer’s instructions.

### In vitro osteogenic differentiation assay

PDLSCs were grown in mineralization-inducing media containing 100 μM/ml ascorbic acid, 2 mM β-glycerophosphate, and 10 nM dexamethasone. For detecting mineralization, cells were induced for 3 weeks, fixed with 70% ethanol, and stained with 2% Alizarin Red (Sigma-Aldrich). Calcium nodule areas were quantified using NIH ImageJ. After Alizarin Red staining, the relative concentration of calcium was measured after extracting dye with 10% cetylpyridinium chloride (CPC; Sigma-Aldrich) for 1 h. Absorbance values were measured in a microplate reader (Bio-Rad Labs) at 562 nm. The relative concentration of calcium was calculated against a standard curve.

### In vitro adipogenic differentiation assay

For adipogenic induction, a StemPro® Adipogenesis Differentiation Kit (Invitrogen, USA) was used. Four weeks after induction, cultured cells were stained with Oil Red O, and positive cells were quantified using NIH ImageJ.

### Reverse transcription polymerase chain reaction (RT-PCR)

Total RNA was isolated from PDLSCs using Trizol reagent (Invitrogen, USA). For real-time RT-PCR, cDNA was synthesized from 2 μg RNA using random hexamers or oligo dT and reverse transcriptase according to the manufacturer’s protocol (Invitrogen, USA). Real-time PCR reactions were performed using the QuantiTect SYBR Green PCR kit (Qiagen, Germany) and iCycler iQ Multi-color Real-time PCR Detection System. The specific primers used for RT-PCR are listed in Additional file 1: (Table S1).

### Measurement of NO

Cell culture supernatants were collected for measurement of NO levels. NO levels were measured using a Griess Reagent kit (Beyotime) according to the manufacturer’s instructions. Absorbance values were measured in a microplate reader (Bio-Rad Laboratories) at 540 nm. SNP (75 μM, Sigma-Aldrich) was used as an NO donor, and l-N^G^-monomethyl arginine (l-NMMA; 1 mM, Sigma-Aldrich) was used as an NO inhibitor. The NO concentration was calculated with a standard curve.

### Immunofluorescent staining

Cells were grown on glass coverslips, fixed in 4% formaldehyde for 10 min, permeabilized with 0.1% Triton X-100 for 5 min, blocked in 10% normal goat serum, and incubated with primary antibodies (1:200) overnight at 4 °C. The samples were then treated with rhodamine/FITC-conjugated secondary antibodies (1:400, Sigma-Aldrich, St. Louis, MO, USA) and mounted with Vectashield mounting medium containing 4’,6-diamidino-2-phenylindole (DAPI; Sigma-Aldrich). Images were captured with a confocal microscope (AX10, Carl Zeiss, Gottingen, Germany).

### Statistical analysis

All statistical calculations were performed using SPSS 18.0 statistical software. Student’s *t* test or one-way analysis of variance (ANOVA) were performed to determine statistical significance (*P* < 0.05).

## Results

### iNOS and eNOS expression and NO production in PDLSCs

**Fig. 1:**
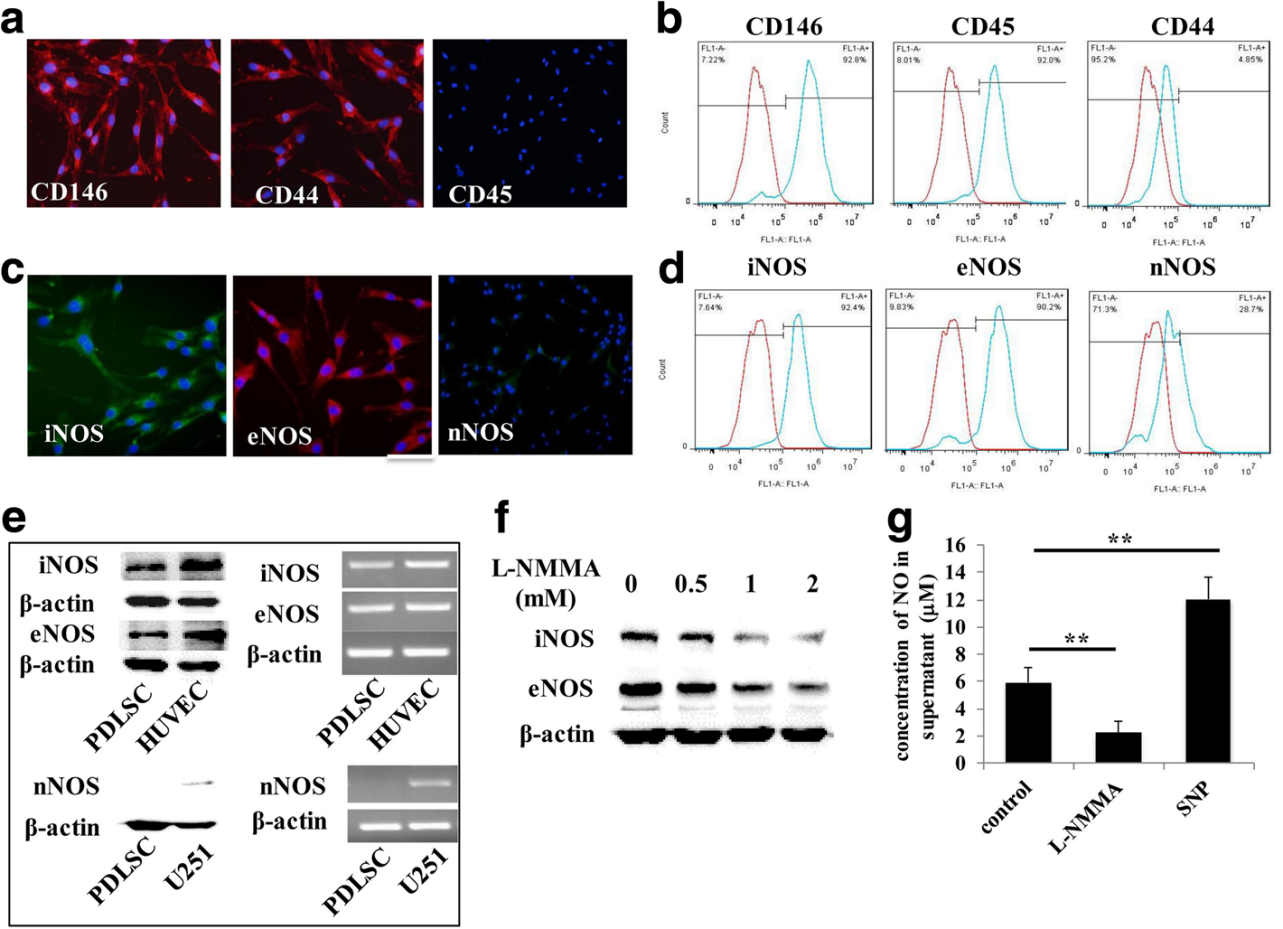
Nitric oxide (NO) and its synthesizing enzymes inducible NO synthase (iNOS) and endothelial NO synthase (eNOS) are expressed in periodontal ligament stem cells (PDLSCs). **a,b** Immunofluorescent staining and flow cytometry show that cells used in this study express CD146 and CD44 but not CD45. **c–e** Western blot, RT-PCR, immunofluorescent staining, and flow cytometry show that PDLSCs express iNOS and eNOS. Human umbilical vein endothelial cells (HUVECs) were used as positive control. Scale bar = 50 μm. **f** PDLSCs were treated with the NOS inhibitor l-N^G^-monomethyl arginine (l-NMMA) for 6 h. Western blotting shows that 1 mM l-NMMA significantly inhibits iNOS and eNOS expression. **g** NO was detectable in PDLSC culture supernatant and was significantly downregulated by l-NMMA and upregulated by sodium nitroprusside (SNP). nNOS, neuronal nitric oxide synthase

We first identified whether all three isoforms of NOS were expressed in PDLSCs. Since both iNOS and eNOS have been reported to be expressed in human umbilical vein endothelial cells (HUVECs) [13] and nNOS is expressed in glioma cell (U251) [14], we used HUVECs and U251 as a positive control. We found that PDLSCs highly expressed iNOS and eNOS but very little nNOS, as shown by Western blot, immunofluorescent staining, and flow cytometry (Fig. 1c–e). Moreover, the NOS inhibitor l-NMMA significantly downregulated the expression of iNOS/eNOS (Fig. 1f). Considering that a high dose of inhibitor may be cytotoxic, we used 1 mM l-NMMA in subsequent experiments. We next showed that PDLSCs produced 6–8 μM NO in culture supernatants, and the levels of NO were significantly downregulated and upregulated by l-NMMA and NO donor SNP, respectively (Fig. 1g).

### Blocking the production of NO has no effect on PDLSC proliferation and apoptosis

**Fig. 2:**
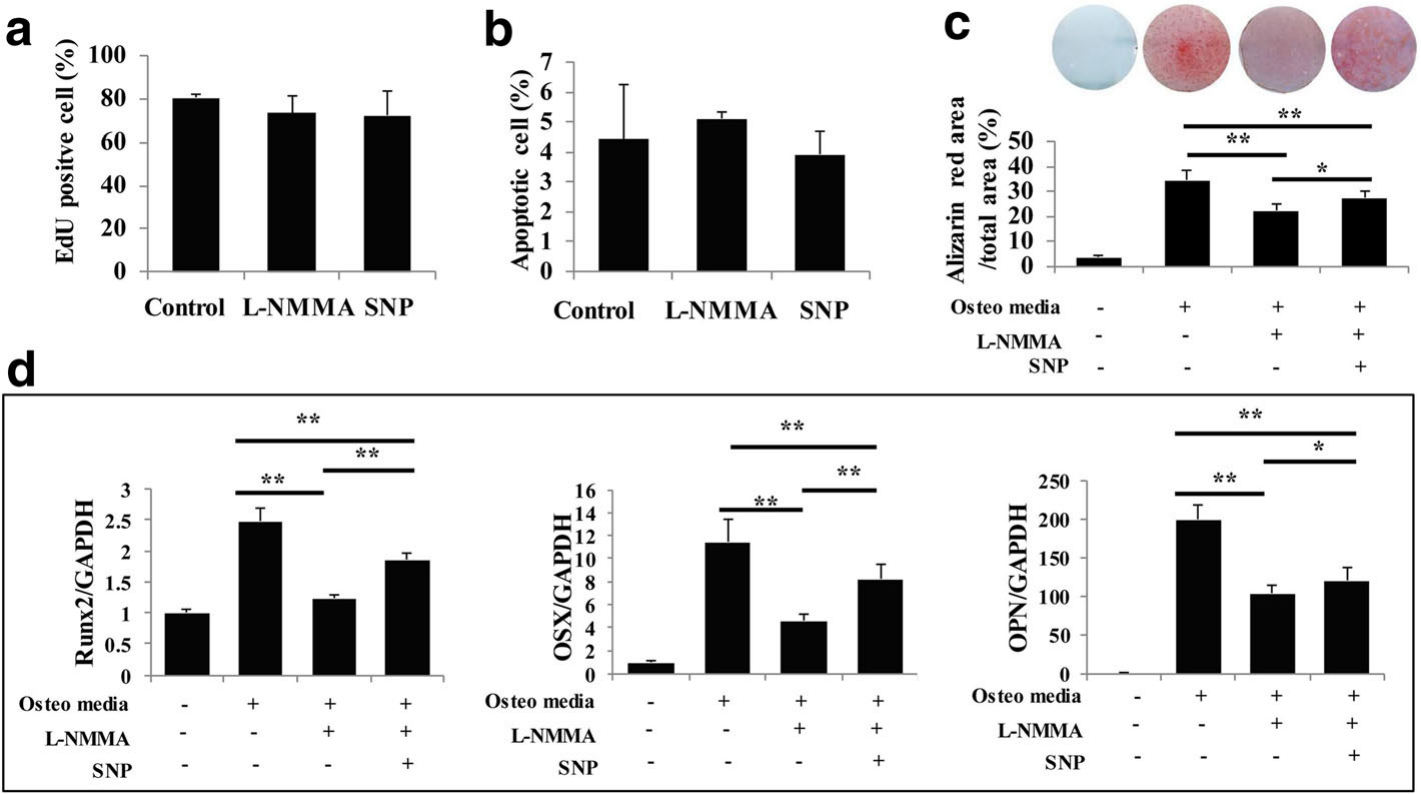
NO has no influence on PDLSC proliferation and apoptosis, while physiological levels of NO are necessary for the mineralization of PDLSCs. **a** When NO generation was blocked by l-N^G^-monomethyl arginine (l-NMMA), there was no change in the proliferation capacity of PDLSCs, as evidenced by EdU assay. **b** Flow cytometric analysis shows that l-NMMA had no effect on PDLSC apoptosis. **c** Alizarin Red staining shows that l-NMMA (1 mM) treatment significantly reduced the formation of mineralized nodules in PDLSCs. Data are representative of three independent experiments. *P < 0.05, **P < 0.01. **d** Real-time RT-PCR showing that l-NMMA treatment downregulated the expression of runt-related transcription factor 2 (Runx2), osterix (OSX), and osteopontin (OPN) mRNA, while NO donor sodium nitroprusside (SNP) treatment largely rescued the expression of these osteogenic markers. All experiments are representative of three replicates. *P < 0.05, **P < 0.01

To analyze the effects of NO on PDLSC proliferation and apoptosis, we added l-NMMA and SNP to PDLSCs and then performed cell proliferation and apoptosis assays. EdU and apoptosis assays showed that reduction of NO or increasing the physiological concentration of NO had no influence on PDLSC proliferation and apoptosis (Fig. 2a, b).

### A physiological level of NO is necessary for the mineralization ability of PDLSCs

Next, to examine whether NO affected the osteogenic potential of PDLSCs, cells were treated with l-NMMA or without l-NMMA . Three weeks after osteogenic induction, Alizarin Red staining revealed that mineralization was significantly lower in l-NMMA-treated cells than in osteogenic-inducing medium-treated cells, and SNP partially rescued the impaired osteogenic potential (Fig. 2c). Consistently, real-time RT-PCR results showed that the expression levels of the osteogenic markers osteopontin (OPN), runt-related transcription factor 2 (Runx2), and osterix (OSX) were significantly reduced in l-NMMA-treated cells (Fig. 2d). SNP treatment significantly rescued the expression of these osteogenic markers (Fig. 2d).

### The adipogenesis capacity of PDLSCs is enhanced when blocking endogenous NO production

**Fig. 3:**
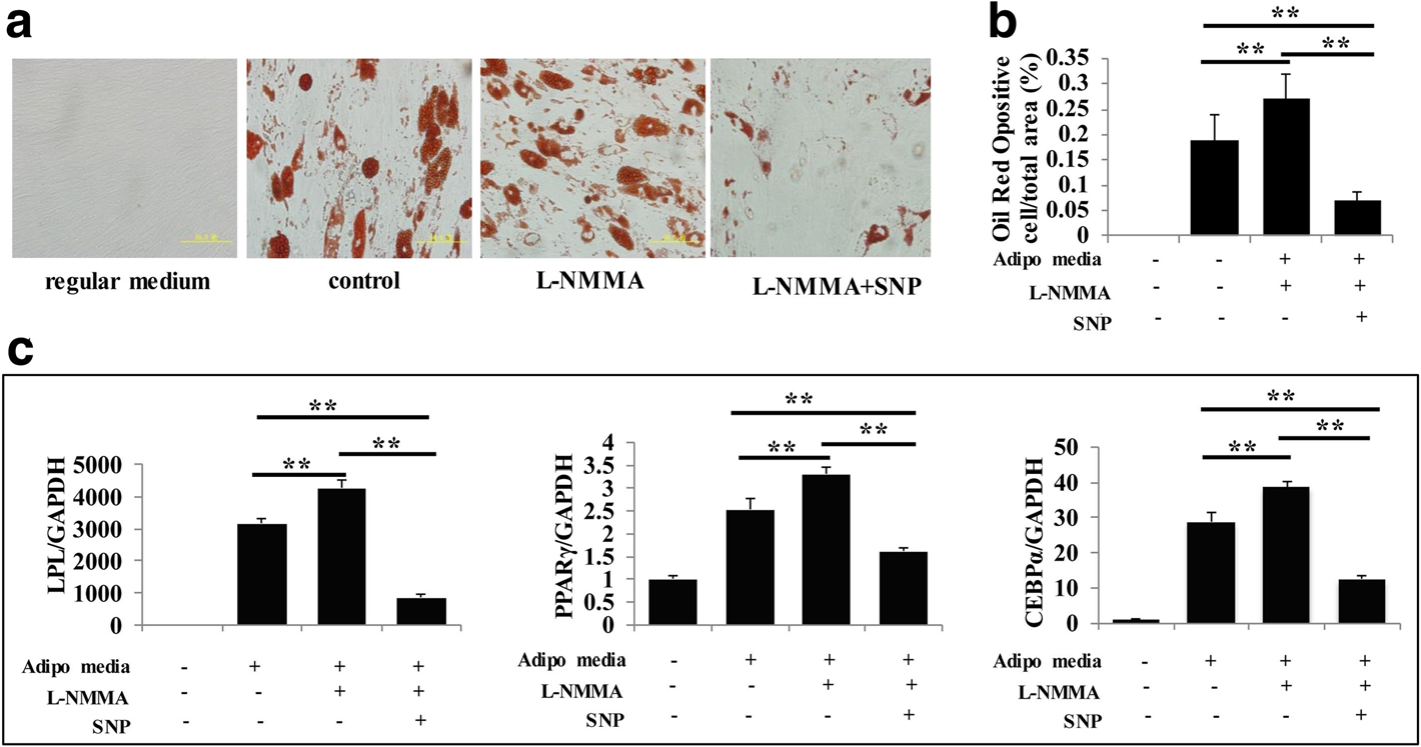
The adipogenic capacity of PDLSCs is enhanced when endogenous NO production is blocked. **a** Four weeks after induction of adipogenic differentiation, PDLSCs were stained with Oil Red O. Data are representative of three independent experiments. Scale bar = 50 μm. **b** ImageJ semiquantitative analysis showing that l-N^G^-monomethyl arginine (l-NMMA) significantly induced cellular lipid accumulation. This effect was reversed by the NO donor sodium nitroprusside (SNP). **P < 0.01. **c** Real-time RT-PCR analysis of gene expression during adipogenic differentiation. Lipoprotein lipase (LPL), peroxisome proliferator-activated receptor (PPAR)γ, and CCAAT-enhancer binding protein (C/EBP)α mRNA levels were upregulated in PDLSCs treated with l-NMMA, and NO donor SNP reduced the upregulation of these genes. **P < 0.01

We next investigated the effect of NO on the adipogenesis of PDLSCs. After 4 weeks of adipogenic induction, cells were treated with l-NMMA resulting in adipogenic conversion as indicated by increased cellular lipid accumulation revealed by Oil Red O staining, and this phenotype was reversed by the NO donor SNP (Fig. 3a, b). In parallel, we evaluated the expression of adipogenesis-induced genes, including those encoding the late marker lipoprotein lipase (LPL) and the transcription factors peroxisome proliferator-activated receptor (PPAR)γ2 and CCAAT-enhancer binding protein (C/EBP)α. Consistent with the Oil Red O staining, l-NMMA stimulated the expression of adipogenic markers, while NO donor reduced the upregulation of these genes (Fig. 3c).

### NO balances the osteogenic and adipogenic potential of PDLSCs through the JNK/MAPK signaling pathway

**Fig. 4:**
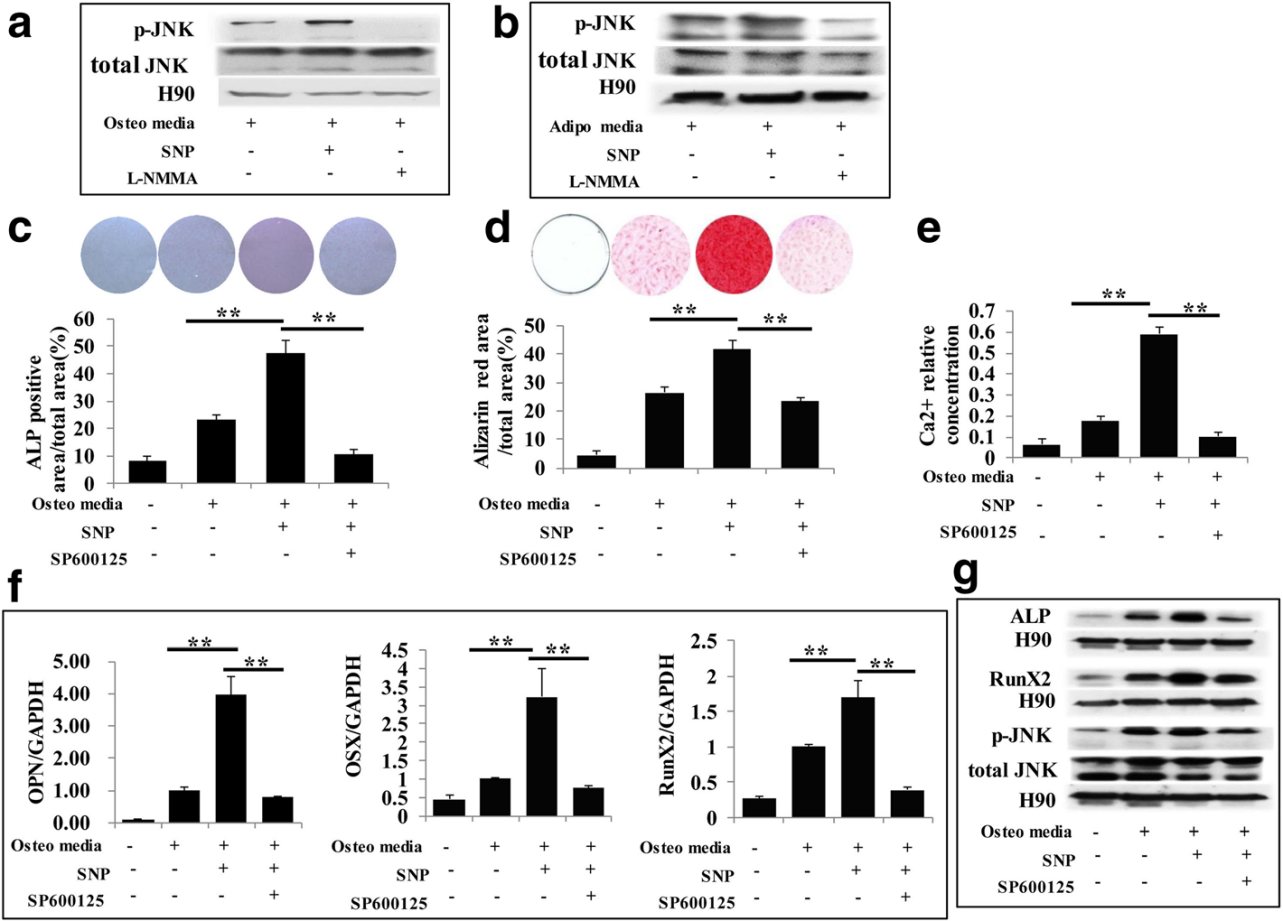
NO promoted the osteogenic capacity of PDLSCs through the JNK/MAPK pathway. **a,b** Western blot showing that phosphorylated c-Jun N-terminal kinase (p-JNK) was decreased when NO generation was blocked by l-N^G^-monomethyl arginine (l-NMMA) during osteogenic or adipogenic induction, while sodium nitroprusside (SNP) significantly increased the expression of p-JNK. **c** Alkaline phosphatase (ALP) staining showing that SNP treatment upregulated the level of ALP, while blocking the JNK/MAPK signaling pathway restrained this SNP-induced ALP expression. **P < 0.01. **d** Alizarin Red staining showed that SNP treatment significantly increased the formation of mineralized nodules in PDLSCs, and this effect was reversed by inhibiting JNK/MAPK. Data are representative of three independent experiments. **P < 0.01. **e** Calcium concentration measurement showing that blocking the JNK/MAPK signaling pathway reduces SNP-induced calcium levels. **P < 0.01. **f** Real-time RT-PCR analysis of osteogenic marker expression during differentiation. Runt-related transcription factor 2 (Runx2), osterix (OSX), and osteopontin (OPN) mRNA levels were significantly increased by SNP, while JNK inhibitor partially eliminated the influence of SNP. All experiments are representative of three replicates. **P < 0.01. **g** Western blot showing that SNP treatment upregulated ALP, Runx2, and p-JNK, and JNK inhibitor treatment significantly reduced this upregulation

**Fig. 5:**
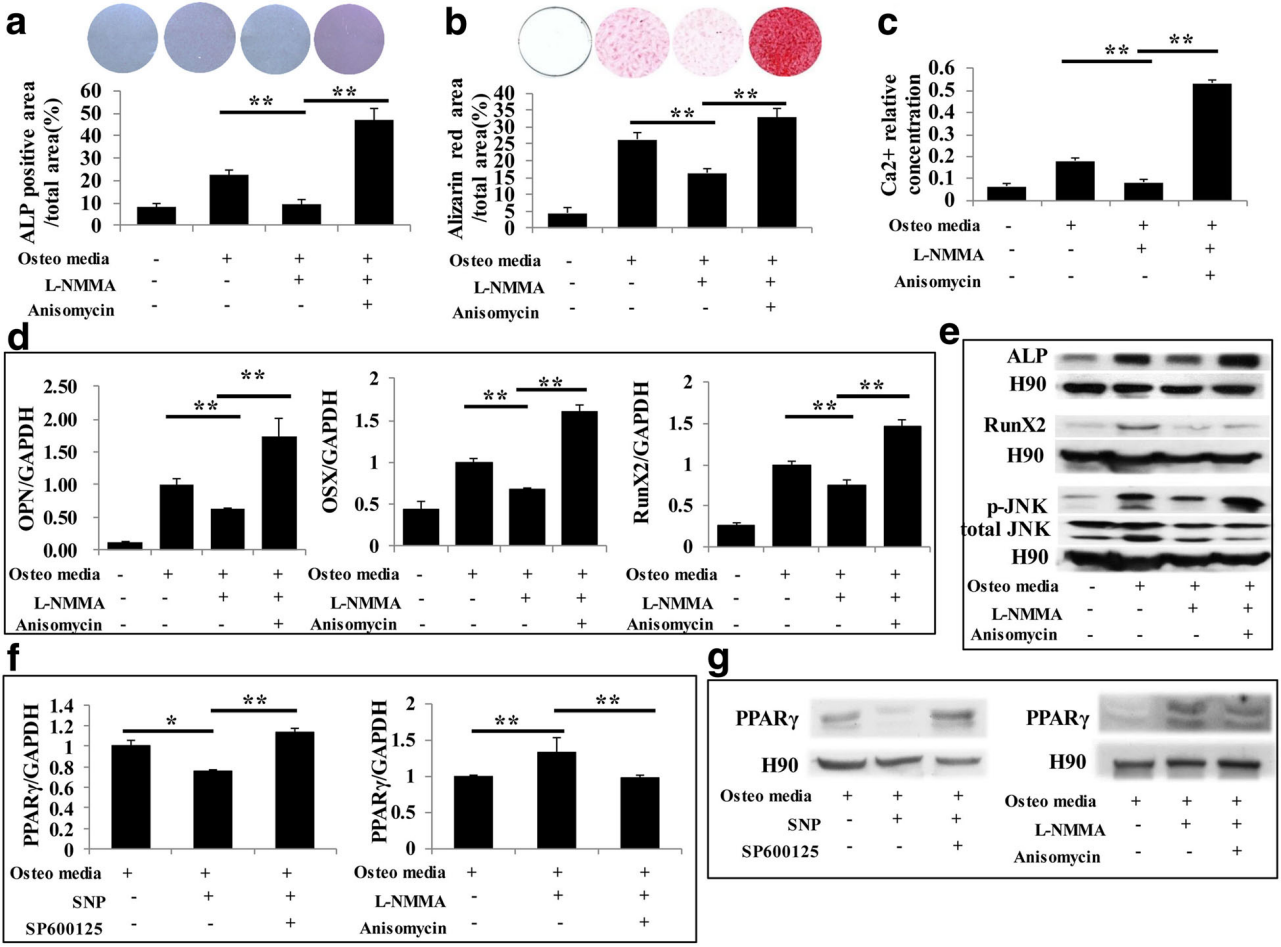
Decreased osteogenic capacity of PDLSCs by inhibiting NO production could be rescued by activating the JNK/MAPK pathway. **a** Alkaline phosphatase (ALP) staining showing that l-N^G^-monomethyl arginine (l-NMMA) treatment downregulated the level of ALP, while activating the JNK/MAPK signaling pathway rescued its expression. **b** Alizarin Red staining showing that l-NMMA significantly reduced mineralization, and this effect was reversed by activating JNK/MAPK. Data are representative of three independent experiments. **P < 0.01. **c** Activating the JNK/MAPK signaling pathway restored calcium levels downregulated by l-NMMA. **P < 0.01. **d** Real-time RT-PCR showing that l-NMMA treatment significantly reduced runt-related transcription factor 2 (Runx2), osterix (OSX), and osteopontin (OPN) mRNA expression levels, while activating the JNK/MAPK pathway largely eliminated the downregulation of osteogenic markers caused by l-NMMA. All experiments are representative of three replicates. **P < 0.01. **e** Western blot showing that l-NMMA downregulated the expression of ALP, Runx2, and phosphorylated c-Jun N-terminal kinase (p-JNK), while JNK activator largely restored expression of these markers. **f,g** Real time RT-PCR and Western blot analysis of adipogenic marker expression during osteogenic differentiation. The peroxisome proliferator-activated receptor (PPAR)γ level was significantly reduced by sodium nitroprusside (SNP), while SP600125 partially eliminated this effect. Blocking NO generation with l-NMMA led to significant upregulation of PPARγ, and anisomycin crippled this influence. All experiments are representative of three replicates. **P < 0.01

We next investigated the possible signaling pathway involved in the differentiation of PDLSCs induced by NO. The MAPK family includes extracellular signal-regulated kinase (ERK), P38 kinase, and JNK, which are involved in regulating numerous cell functions, such as differentiation, proliferation, and apoptosis [15]. There is substantial evidence that MAPK signaling plays an important role in regulating cell differentiation [16]. We found that NO enhanced the phosphorylation of JNK during osteogenesis and adipogenesis, while blocking NO production led to the opposite result (Fig. 4a, b). Based on this result, further experiments were performed for verification purposes. ALP staining was applied on the fourth day after osteogenic induction, showing that SNP effectively promoted osteogenic conversion, and treatment with the JNK signal inhibitor SP600125 slowed this conversion (Figs. 4c and 5a). Furthermore, l-NMMA remarkably inhibited the osteogenic function of PDLSCs, while activating JNK signaling with anisomycin rescued the repression of osteogenesis (Fig. 4c and Fig. 5a). After 3 weeks of osteogenic induction, Alizarin Red staining showed that SNP treatment promoted osteogenic conversion as shown by the increase in cellular calcium nodules, as well as elevated calcium concentrations. These phenotypes were reversed by treatment with the JNK inhibitor SP600125 (Fig. 4d, e and Fig. 5b, c). Blocking NO production with l-NMMA led to a significant decrease in PDLSC mineralization, while the JNK activator anisomycin partially rescued the impairment of osteogenesis (Fig. 4d, e and Fig. 5b, c). Meanwhile, real-time RT-PCR and Western blot results showed that the expression levels of the osteogenic markers OPN, OSX, Runx2, and ALP were significantly increased in NO-treated cells, while the JNK inhibitor reduced their induction (Fig. 4f, g and Fig. 5d, e). Anisomycin reversed the repression of osteogenic markers caused by l-NMMA (Fig. 4f, g and Fig. 5d, e). Furthermore, we discovered that SNP and anisomycin significantly reduced the expression of the adipogenesis-related transcription factor PPARγ2 during the PDLSC osteogenesis process, while l-NMMA and SP600125 led to the opposite result (Fig. 5f). Consistent with previous results (Fig. 4a, b), p-JNK expression, as measured by Western blot, was increased by SNP and decreased by l-NMMA (Figs. 4g and 5e).

**Fig. 6:**
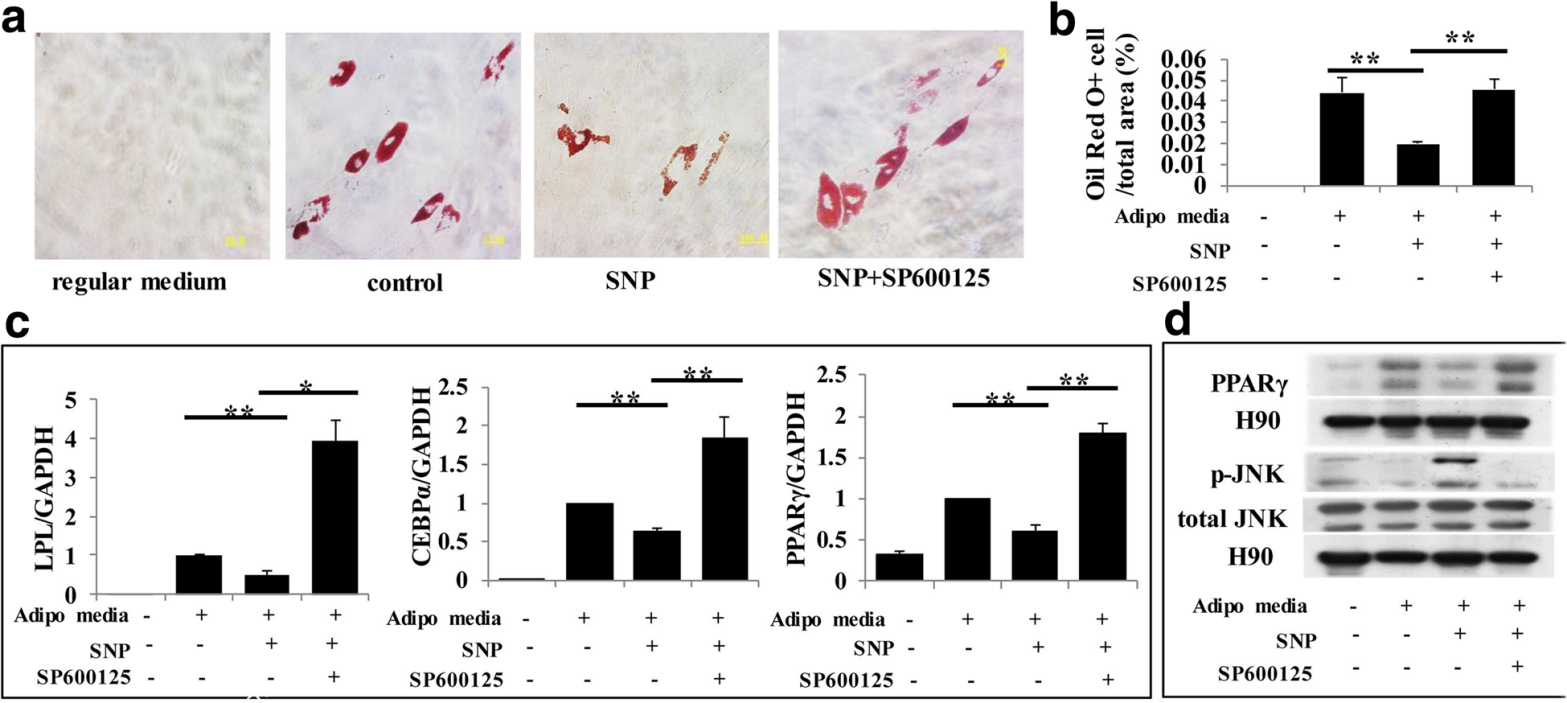
NO inhibited the adipogenic capacity of PDLSCs through the JNK/MAPK pathway. **a,b** Oil Red O staining indicating that sodium nitroprusside (SNP) treatment downregulated the level of adipogenesis, while blocking the JNK/MAPK signaling pathway reversed this effect. Data are representative of three independent experiments. **P < 0.01. **c** Real-time RT-PCR showing that l-NMMA treatment induced the expression of lipoprotein lipase (LPL), peroxisome proliferator-activated receptor (PPAR)γ, and CCAAT-enhancer binding protein (C/EBP)α mRNA, but activating the JNK/MAPK pathway largely reversed their upregulation. All experiments are representative of three replicates. **P < 0.01. **d** Western blot showing that SNP treatment repressed the expression of PPARγ and phosphorylated c-Jun N-terminal kinase (p-JNK), while JNK inhibitor treatment enhanced the expression of these markers

**Fig. 7:**
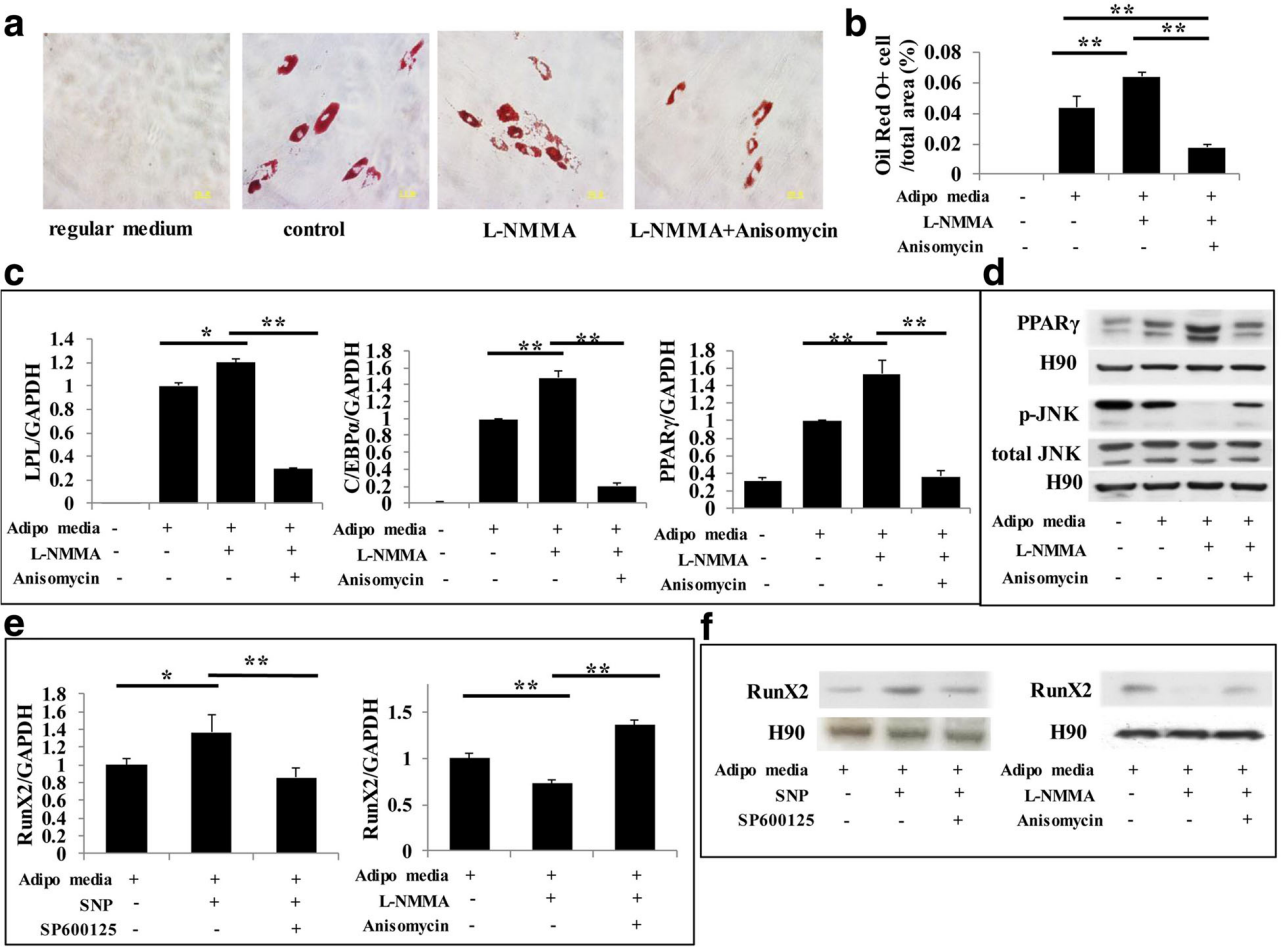
Increased adipogenic capacity of PDLSCs by inhibiting NO production could be reversed by activating the JNK/MAPK pathway. **a,b** Oil Red O staining indicating that lipid accumulation was significantly increased by l-N^G^-monomethyl arginine (l-NMMA), while this effect was reversed by a JNK/MAPK activator. Data are representative of three independent experiments. **P < 0.01. **c** Real time RT-PCR showing that lipoprotein lipase (LPL), peroxisome proliferator-activated receptor (PPAR)γ, and CCAAT-enhancer binding protein (C/EBP)α mRNA levels were downregulated in PDLSCs treated with sodium nitroprusside (SNP), while JNK inhibitor rescued the JNK/MAPK downregulation of these genes. All experiments are representative of three replicates. **P < 0.01. **d** Western blot indicating that PPARγ and phosphorylated c-Jun N-terminal kinase (p-JNK) levels were significantly increased by l-NMMA, while the JNK activator could reduce the upregulation of these markers. **e,f** Real-time RT-PCR and Western blot showing that the expression level of the osteogenic marker runt-related transcription factor 2 (Runx2) was significantly increased by SNP during adipogenic differentiation, while SP600125 could partially eliminate this effect. While blocking NO generation by l-NMMA led to downregulation of Runx2, anisomycin reversed this influence. All experiments are representative of three replicates. **P < 0.01

To investigate adipogenesis, cells were cultured with adipogenesis-inducing medium for 4 weeks. We found that SNP-treated PDLSCs significantly resisted differentiation into adipocytes, which was confirmed by Oil Red O staining. This decreased adipogenic conversion was partially rescued by blocking the JNK signaling pathway (Fig. 6a, b and Fig. 7a, b). In contrast, l-NMMA caused an elevated level of adipogenic conversion compared with the control group, while activating the JNK pathway partially reduced the effect (Fig. 6a, b and Fig. 7a, b). These phenomena were further confirmed with real-time RT-PCR and Western blots for adipogenesis-induced markers including LPL, PPARγ, and C/EBPα, and the results were consistent with Oil Red O staining (Fig. 6c, d and Fig. 7c, d). During induction of adipogenesis, the expression of the osteogenic marker Runx2 was significantly reduced by blocking NO production but increased by SNP treatment (Fig. 7e). While the effect of SNP was reversed by inhibiting the JNK signaling pathway, the effect of l-NMMA was reversed by activating the JNK signaling pathway (Fig. 7f).

Moreover, consistent with previous results (Fig. 4a, b), Western blots showed that p-JNK levels were increased by SNP and decreased by l-NMMA during the adipogenic process (Fig. 4g, Fig. 5e, Fig. 6d, and Fig. 7d). This result further confirmed the molecular mechanism of NO-induced PDLSC differentiation.

**Fig. 8:**
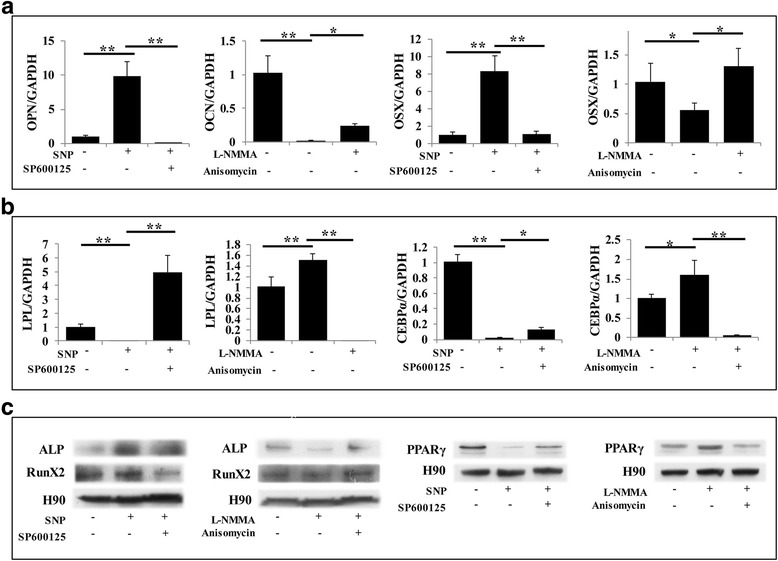
NO and the JNK signaling pathway work to the same effect in regular medium compared with induced medium. **a** Real-time RT-PCR showing that the expression level of osteopontin (OPN) and osterix (OSX) were significantly increased by sodium nitroprusside (SNP) in PDLSCs in regular medium, while SP600125 could eliminate this effect. Blocking NO generation by l-N^G^-monomethyl arginine (l-NMMA) in PDLSCs in regular medium led to downregulation of OPN and OSX, and anisomycin could reverse this influence. All experiments are representative of three replicates. **P < 0.01. **b** Real-time RT-PCR showing that the expression level of lipoprotein lipase (LPL) and CCAAT-enhancer binding protein (C/EBP)α were significantly decreased by SNP in PDLSCs in regular medium, while SP600125 could eliminate this effect. Blocking NO generation by l-NMMA in PDLSCs in regular medium led to upregulation of LPL and C/EBPα, and anisomycin could reverse this influence. All experiments are representative of three replicates. **P < 0.01. **c** Western blot indicating that alkaline phosphatase (ALP) and runt-related transcription factor 2 (Runx2) levels were significantly increased by SNP in PDLSCs in regular medium, while the expression of peroxisome proliferator-activated receptor (PPAR)γ was inhibited. Blocking the JNK signaling pathway was able to reverse the influence induced by NO. When the NO level was downregulated by l-N^G^-monomethyl arginine (l-NMMA) in PDLSCs in regular medium, the expression of ALP and Runx2 were downregulated and PPARγ was upregulated. Activating the JNK signaling pathway was able to reverse the influence of l-NMMA

We also studied the effects of NO and JNK on PDLSCs in regular (standard) medium to verify the above conclusions. From the results of real time RT-PCR and Western blot, we found that NO and JNK had the same effect in regular medium compared with induced medium. NO could induce upregulation of the expression of the osteogenic markers ALP, Runx2, OPN, and OSX, and reduced the expression of the adipogenic markers LPL, PPARγ and C/EBPα. When blocking the JNK signaling pathway, the NO-induced increase in osteogenic markers was inhibited and adipogenic markers were upregulated; activating the JNK signaling pathway produced opposite effect (Fig. 8a–c). Thus, we infer that JNK plays a major role in balancing the osteogenic and adipogenic differentiation of PDLSCs under the effects of NO.

## Discussion

PDLSCs are a type of mesenchymal stem cell (MSC) with strong potential for proliferation and multipotent differentiation. MSC lineage differentiation can be regulated at different molecular levels [17, 18]. The shift between osteoblastic and adipocyte lineages is a result of crosstalk between various factors that drive MSCs toward the adipocyte lineage, inhibiting osteoblast differentiation [19, 20]. Since osteoblasts and adipocytes share a common origin, a switching mechanism in MSCs is important for regenerative medicine. In the present study, we show that decreasing endogenous NO production with a NOS inhibitor increases PDLSC-mediated adipocyte differentiation while reducing the number of osteoblasts. In contrast, the NO donor SNP reversed the effect of NOS inhibition, suggesting that endogenous NO is essential for maintaining the balance between osteoblasts and adipocytes in PDLSCs.

It has been suggested that NO mediates the effects of physical activity on bones, including bone development, bone healing, and bone resorption [21–24]. Mice lacking eNOS exhibit profound abnormalities in bone formation, and osteoblasts isolated from eNOS-null mice show significant delays in differentiation and a reduction in Runx2 levels, suggesting that NO regulates Runx2 expression [25, 26]. Treatment of eNOS-null osteoblastic cells with NO donors significantly rescued the levels of Runx2 and was correlated with an enhancement of cell differentiation [26, 27]. The contribution of NO in MSC osteogenesis has also been reported. Increased NO production was previously observed in human PDLSCs during their osteogenic differentiation [9]; however, in that case, a causal relationship was not demonstrated. The results of the present study show that, when treated with the NOS inhibitor l-NMMA to reduce NO levels, PDLSCs showed a reduced capacity for forming mineralized nodules in vitro, with downregulation of Runx2, OSX, and OPN. These observations suggest that NO is required to maintain PDLSC osteogenic differentiation.

Observations concerning the role of NO in adipocyte differentiation have remained controversial. Several studies have reported that the production of NO promotes adipocyte differentiation [28–30]. Conversely, results from other studies have suggested that NO may have the opposite effect on adipogenesis [31–33]. Among these studies, one focused on the role of NO in the adipogenesis of mesenchymal tissue-derived progenitors [33]. NO has been shown to inhibit the adipogenesis of mesenchymal fibro-adipogenic progenitors by inducing expression of miR-27b and downregulating PPARγ. In the present study, we found that the NOS inhibitor l-NMMA increased the number of Oil Red O-positive cells and enhanced expression levels of LPL, PPARγ, and CEBP/α in PDLSCs, while NO donor treatment resulted in a significant reduction in PDLSC adipogenesis. Determining whether NO acts as a pro-adipogenic or anti-adipogenic factor requires further research.

The MAPK signaling pathway plays vital roles in maintaining cell physiological function, and the present study confirmed that this signaling pathway can induce cell differentiation. Research concerning the role of the JNK signaling pathway in adipocyte differentiation has remained controversial. Several studies have reported that JNK activity is specifically required for the initial stage of differentiation events of adipocytes and may act with a positive impact in adipogenesis differentiation [34, 35]. Conversely, results from other studies have suggested that JNK activity may have the opposite effect on adipogenesis [36–38]. In our study, we found that, in both adipogenic and osteogenic differentiation processes, NO increased the phosphorylation of JNK/MAPK, and then p-JNK transportation to the nucleus induced the expression of osteogenic transcription factors and repressed the expression of adipogenic transcription factors, thus increasing osteogenesis and reducing adipogenesis. Conversely, blocking NO production in PDLSCs led to a decrease in phosphorylation of JNK/MAPK with the opposite differentiation result. These results strongly suggest that JNK/MAPK acts as a switch in NO-induced cellular differentiation of PDLSCs. It is hard to explain the inconsistent effect of JNK in the adipogenesis process between our results and other studies [34, 35], and we suggest the consideration that the influence of NO and cell type may regulate the function of adipogenesis. So far, the effects of NO and JNK on stem cell adipogenesis remains controversial. In addition, the components upstream of JNK/MAPK in NO-induced PDLSC differentiation are still unclear. Several reports showed that PPARγ may act upstream of JNK activation or inhibit the JNK downstream target, AP-1, to regulate cell functions. AP-1 is a heterodimer consisting of c-fos (Fra-1, Fra-2, c-Fos, FosB) and c-jun (c-Jun, JunB, JunD) and acts as a major transcription activator in cells, controlling many cellular processes. AP-1 is recognized as a JNK downstream target, activated by the JNK signaling pathway and promoting downstream gene expression to regular cell functions; it is reported to be involved in cell inflammation, apoptosis, and osteogenesis differentiation [39, 40]. In the process of PDLSC differentiation, PPARγ may block AP-1 which, combined with targeted DNA or competitive binding with CBP to inhibit AP-1 activation, leads to downregulation of osteogenesis with increased adipogenesis. Blocking the JNK signaling pathway increased the expression of PPARγ and further decreased AP-1 activity, which induced adipogenesis differentiation of PDLSCs [39, 40]. In our study, we focused on the switch effect of JNK in NO-induced PDLSC differentiation, and the deeper molecular mechanism of NO-induced adipogenesis differentiation requires further research.

In previous studies, Runx2 and PPARγ have been shown to be vital osteogenic and adipogenic transcription factors, respectively, playing major roles in stem cell differentiation [41]. In our study, NO activated the JNK/MAPK signaling pathway during the differentiation process, thus increasing the phosphorylation level of JNK. Subsequently, p-JNK was transported to the nucleus, where it promoted Runx2 transcription activity through phosphorylation, inducing higher expression of the transcription factor Runx2 and ultimately accelerating the osteogenesis of PDLSCs. On the other hand, NO reduced the transcriptional activity of PPARγ through the JNK signaling pathway, downregulating PPARγ expression and thus suppressing the adipogenic conversion of PDLSCs. When NO generation was blocked during osteogenic differentiation, p-JNK was downregulated leading to a lower expression of Runx2 but elevated levels of PPARγ, inhibiting osteogenic differentiation while promoting adipogenic conversion. Our results further confirm that NO balances the osteogenic and adipogenic differentiation of PDLSCs by regulating the expression of Runx2 and PPARγ transcription factors through the JNK/MAPK pathway.

## Conclusion

In conclusion, the results of this study demonstrate that blocking the production of NO in PDLSCs downregulated JNK/MAPK, thus inhibiting osteogenesis while increasing adipogenesis. In contrast, the addition of NO promotes osteogenesis by upregulating JNK/MAPK and reducing adipogenesis. NO is essential for maintaining the balance between osteoblasts and adipocytes in PDLSCs through JNK/MAPK signaling. These findings may be important for our understanding and clinical application of stem cell therapy.

## Additional file

Additional file 1: **Table S1**. List of the specific primers used for RT-PCR. (JPG 58 kb)
