# Supplementary figures and images for: Nitric oxide balances osteoblast and adipocyte lineage differentiation via the JNK/MAPK signaling pathway in periodontal ligament stem cells
Source: Stem Cell Res Ther. 2018 May 2;9:118. doi: 10.1186/s13287-018-0869-2 (PMC5930947; doi:10.1186/s13287-018-0869-2)

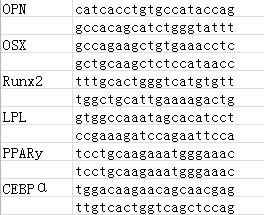

Supplement: Supplementary file 1 — Table S1. List of the specific primers used for RT-PCR. (JPG 58 kb) [file 13287_2018_869_MOESM1_ESM.jpg]
